# Association between Low blood lead levels and increased risk of dental caries in children: a cross-sectional study

**DOI:** 10.1186/s12903-017-0335-z

**Published:** 2017-01-13

**Authors:** Young-Suk Kim, Mina Ha, Ho-Jang Kwon, Hae-Young Kim, Youn-Hee Choi

**Affiliations:** 1 Department of Dental Hygiene, U1 University, Yeongdong, Korea; 2 Department of Preventive Medicine, Dankook University College of Medicine, 119 Dandae-ro, Dongnam-gu, Cheonan, Chungnam 31116 Republic of Korea; 3 Department of Health Policy and Management, College of Health Science, and Department of Health Science, Graduate School, Korea University, Seoul, Korea; 4 Department of Preventive Dentistry, School of Dentistry, Kyungpook National University, Daegu, Korea

**Keywords:** Blood lead, Children, Dental caries, Deciduous dentition

## Abstract

**Background:**

The objective of this study was to examine the association between low blood lead levels of <5 μg/dL and the development of dental caries among children.

**Methods:**

The Children’s Health and Environment Research (CHEER) group recruited a cohort of 7,059 school-aged children from six Korean cities. The final study populations in the permanent and deciduous teeth groups were 1,564 and 1,241 children, respectively, after excluding 4 children with blood lead levels of >5 μg/dL. Compared with the children who did not have dental caries, the risk of having dental caries according to blood lead level was estimated by using the zero-inflated negative binomial model.

**Results:**

The geometric mean (geometric standard deviation, maximum) blood lead level was 1.53 μg/dL (1.57, 4.89 μg/dL), and 74.4% of children had a level of <2 μg/dL. Blood lead level was significantly higher in the children with than in those without deciduous dental caries (1.59 vs. 1.51 μg/dL), similarly with permanent dental caries (1.65 vs. 1.51 μg/dL). After adjustment for covariates, deciduous teeth surfaces that were decayed and filled increased significantly with increasing blood lead levels in a dose-dependent manner (prevalence ratio, 1.14; 95% confidence interval: 1.02–1.27). However, the risk of having dental caries in permanent teeth was not linearly associated with the increase in blood lead level.

**Conclusions:**

In the sum of decayed and filled surfaces, we found a significant increase in risk of dental caries of the deciduous teeth with an increase in blood lead levels (<5 μg/dL) but found no statistical significance in the association with decayed and filled surfaces of caries separately.

## Background

Dental caries is a chronic disease that progresses slowly in most people [1] and is one of the most prevalent diseases in children worldwide. The prevalence of dental caries among Korean children was 30.4% in 8-year-olds in deciduous teeth and 57.3% in 12-year-olds in permanent teeth in 2012 [2]. In China, the prevalence of dental caries in 2013 was 52% in children aged 5–6 years and 41% in those aged 12–13 years [3]. The proportion of children between the ages of 5 and 17 years who had untreated dental caries was approximately 20.1% in the United States [4].

Dental caries is a complex disease, and several microbial, genetic, immunological, behavioral, and environmental factors contribute to its risk and severity [1]. Dental caries occurs from a series of interactions over time between bacteria, which produce acid, and host factors, including teeth and saliva [1]. Endogenous bacteria produce organic acid that decreases plaque pH, which leads to demineralization of the tooth surface [5, 6]. A destroyed tooth structure requires restoration and maintenance throughout life; therefore, the burden of dental caries continues for a lifetime [1]. Furthermore, it is influenced by various systemic diseases such as asthma [7, 8] and diabetes mellitus [9].

Lead has been used extensively by humans for a long period and spread to the environment widely [10]. In most developed countries, concerted efforts have been made to reduce lead levels in the environment in recent years, although in developing countries, lead is still a significant public health problem [11]. Susceptible populations, including children and pregnant women, may be more affected than non-susceptible populations by environmental lead. The mean blood lead levels were 1.34, 1.26, and 1.14 μg/dL in Korean children aged 3–5, 6–11, and 12–18 years, respectively, from 2012 to 2014 [12], which were higher than the 0.838 and 0.680 μg/dL in US children aged 6–11 and 12–19 years, respectively, from 2009 to 2010 [13]. Higher lead levels in bone and blood are a risk factor of systemic diseases such as ischemic heart disease [14] and hypertension [15]. In addition, blood lead levels affect neurological and neurobehavioral development, as well as oral health.

Each 1-μg/dL increase in blood lead level results in a decrease of 0.87 intelligence quotient (IQ) points; in the range of <10 μg/dL, an increase of 1 μg/dL results in a 1.37 decrease in IQ measured with the Stanford-Binet Intelligence Scale [16]. In an animal study, exposure of less-mature enamel to lead resulted in decreased hardness in comparison with the enamel of control animals [17]. In addition, epidemiological studies have reported an association between lead exposure and dental caries in children [18–22].

However, the blood lead levels in the previous studies were relatively higher (≥2 μg/dL) than those reported recently in children in Korea and other developed countries. The aim of this study was to examine the association between blood lead concentration and dental caries in Korean children with a blood lead level of ≤5 μg/dL, the reference value recently proposed by the US Centers for Disease Control and Prevention (CDC) [23].

## Methods

### Study population

The present study was conducted as a part of the Children’s Health and Environment Research (CHEER) study, which was a cohort study conducted from 2005 to 2010 to investigate the association between environmental exposure and health in school-aged children recruited from urban, rural, and industrial areas within Korea [24]. Children underwent oral examinations from 2008 to 2010 in four cities: Seoul (urban area), Daegu (urban area), Cheonan (rural area), and Busan (industrialized area) at a cross-sectional time frame. After excluding 63 children whose blood lead concentrations were not measured and 4 children who had a blood lead level of >5 μg/dL, the final numbers of subjects in the present study were 1,564 and 1,241 for analysis of permanent and deciduous teeth, respectively. The study protocol was reviewed and approved by the institutional review board of the Dankook University College of Medicine, and all the participants’ parents or guardians were provided written informed consent before participation. This study has been conducted in full accordance with the World Medical Association Declaration of Helsinki.

#### Oral examinations

Oral examinations were performed based on the oral examination guidelines for epidemiological investigation established by the World Health Organization [25]. Two dentists examined the children’s oral status, and a trained dental hygienist recorded the oral index. The oral examination instruments used included a front surface mouth mirror and a double-ended Shepherd’s hook and Orban explorer. Dental caries was defined by the number of decayed surfaces (ds), filled surfaces (fs), and the sum of decayed and filled surfaces (dfs) for deciduous teeth, and by the number of decayed surfaces (DS), filled surfaces (FS), and the sum of decayed and filled surfaces and missing teeth due to dental caries (DMFS) for permanent teeth.

#### Blood lead measurement

To measure blood lead concentrations, 3–5 ml of whole blood was drawn from each child by using a syringe and then sealed in a heparin-containing tube. The blood lead levels were determined by using atomic absorption spectrophotometry (Spectral AA-800 Zeeman Correction, Varian, Australia) at a commercial laboratory. The limit of quantification (LOQ) was 0.030 μg/dL.

#### Potential confounders and covariates

To estimate the risk of dental caries in association with blood lead level, potential confounding factors or covariates were considered. Information on basic demographic variables was obtained from a questionnaire survey, including sex (male or female), age (≤9, 10, 11, and ≥12 years), and mother’s educational level (≤12 and >12 years), and monthly household income (<2000, 2000–2990, 3000–3990, and ≥4000 10^3^ KRW). Urinary cotinine level was measured by using a gas chromatograph-mass selective detector (Perkin Elmer, Clarus 600 T, Waltham, USA), and the limit of detection (LOD) of urinary cotinine was 0.396 μg/dL. We categorized the cotinine level in urine for the analysis as ≤3 or >3 μg/g.

Lead interrupts the absorption of calcium and iron in the human body [10]. Nutritional status of calcium and iron can influence an individual’s susceptibility to lead toxicity [26]. However, blood lead levels were not associated with calcium and iron concentrations in the subjects of the present study. Therefore, calcium and iron concentrations were not included in the multivariate models.

#### Statistical analysis

The Kruskal-Wallis or Wilcoxon rank sum tests were applied to assess differences in geometric mean blood lead level according to sociodemographic characteristics. The adjusted mean blood lead levels for dental caries-positive and dental caries-negative statuses were calculated by using the least square mean estimation in the corresponding multiple linear regression model. Based on a previous study [27], among the Poisson, negative binomial, and zero-inflated Poisson models, the zero-inflated negative binomial model (ZINB) was reported to be the best-fitted model for the dental caries study because of excess zero of caries in children. Therefore, we used the ZINB model adjusted for the above-mentioned potential confounders and covariates. The risk (prevalence ratio) and 95% confidence interval (CI) for having dental caries in deciduous and permanent teeth were estimated. The analyses were conducted by using R version 3.1.1 (R Foundation for Statistical Computing, Vienna, Austria) [28], with a significance level of 0.05. Moreover, Stata 12.1 (Stata Corp LP, College Station, TX, USA) was used to apply the ZINB model.

## Results

In the deciduous teeth group, about 42% of the children had more than one decayed surface (ds) and 73% of the children had more than one filled surface (fs) due to dental caries. Most (81.7%) of the children had more than one dfs in the deciduous teeth group. The number of ds was significantly higher among the males than among the females (*p* = 0.05), but fs and dfs were not significantly different between the sexes in the deciduous teeth group. Geographically, ds, fs, and dfs were significantly higher in the industrial areas than in the urban or rural areas in the deciduous teeth group. The number of ds was significantly higher in the children with less-educated parents, single parents, and lower household income. The prevalence rates of DS, FS, and DMFS were 16.3%, 33.4%, and 44.6%, respectively, in the permanent teeth group. The prevalence rates of FS and DMFS, but not DS, were significantly higher in the females than in the males. As in the deciduous teeth group, DS rate was significantly higher in the children with less-educated parents, single parents, and lower household incomes (Table 1).

**Table 1:** Frequency of dental caries according to general characteristics of study subjects

Characteristics	Deciduous teeth				Permanent teeth			
	N	ds (≥1)	fs (≥1)	dfs (≥1)	N	DS (≥1)	FS (≥1)	DMFS (≥1)
		%	%	%		%	%	%
All	1,241	41.9	72.8	81.7	1,564	16.3	33.4	44.6
Gender								
Boy	646	44.6	74.6	83.1	806	14.9	31.0	41.1
Girl	595	39.0	70.8	80.2	758	17.8	36.0	48.4
*p-value*		*0.05*	*0.14*	*0.20*		*0.14*	*0.04*	*0.004*
Age (years)								
≤ 9	933	42.3	74.3	81.9	963	13.4	29.7	39.6
10	62	37.1	64.5	77.4	92	16.3	34.8	44.6
11	177	43.5	70.1	83.1	300	22.0	39.3	53.0
≥ 12	69	36.2	66.7	79.7	209	21.5	41.6	56.0
Mean (SD)	9.8 (1.1)				10.2 (1.3)			
*p-value*		*0.62*	*0.16*	*0.76*		*0.001*	*0.001*	*<0.0001*
Living area								
Urban (Seoul,Daegu)	757	38.6	73.4	80.6	986	14.4	35.0	44.1
Industrial (Busan)	216	54.2	78.7	88.4	226	21.7	39.4	55.8
Rural (Cheonan)	268	41.4	66.0	79.5	352	18.2	25.3	38.9
*p-value*		*0.0002*	*0.01*	*0.02*		*0.02*	*0.001*	*0.0003*
Educational level of father (years)								
≤ 12	436	48.4	73.2	83.5	572	19.6	34.1	48.6
> 12	641	36.5	73.3	80.2	774	11.8	31.5	38.9
Unknown	164	45.7	69.5	82.9	218	23.9	38.5	54.6
*p-value*		*0.01*	*0.92*	*0.94*		*<0.0001*	*0.56*	*0.05*
Educational level of mother (years)								
≤ 12	583	44.6	73.2	82.5	745	19.9	33.3	47.2
> 12	496	36.9	73.4	80.2	599	9.8	32.1	38.1
Unknown	162	47.5	69.1	83.3	220	21.8	37.7	53.6
*p-value*		*0.19*	*0.99*	*0.97*		*<0.0001*	*0.30*	*0.004*
Parental marital status								
Single	147	52.4	71.4	85.7	200	27.0	33.5	52.5
Couple	1,039	39.6	73.1	80.8	1286	14.0	33.1	42.8
Unknown	55	58.2	70.9	87.3	78	26.9	38.5	55.1
*p-value*		*0.03*	*0.98*	*0.40*		*<0.0001*	*0.84*	*0.08*
Household income (1,000KRW)								
< 2000	248	48.4	71.4	84.7	343	25.4	33.8	51.6
2000–2990	367	41.1	70.0	79.0	452	16.2	35.2	45.4
3000–3990	255	43.5	73.3	81.6	318	12.9	33.0	42.5
≥ 4000	280	35.0	76.8	81.4	333	9.9	32.1	38.7
Unknown	91	44.0	73.6	85.7	118	17.8	30.5	44.1
*p-value*		*0.02*	*0.27*	*0.37*		*0.05*	*0.83*	*0.01*
Urinary cotinine concentration (ug/g cr.)								
≤ 3.0	585	40.9	72.1	81.4	761	16.0	33.2	44.0
> 3.0	346	45.1	76.3	82.7	382	16.8	30.9	41.4
Unknown	310	40.3	70.0	81.3	421	16.4	36.1	48.7
Mean (SD)	2.15 (3.21)				1.93 (3.24)			
*p-value*		*0.23*	*0.19*	*0.68*		*0.82*	*0.46*	*0.43*

*p*-value calculated by *x* ^2^ - test excluding unknown category

*ds* decayed surfaces for deciduous tooth, *fs* filled surfaces because of caries for deciduous tooth, *dfs* the sum of decayed and filled surfaces for deciduous tooth. *DS* decayed surfaces for permanent tooth, *FS* filled surfaces because of caries for permanent tooth, *DMFS* the sum of decayed, filled surfaces and missing because of caries for permanent tooth

The geometric mean blood lead levels (geometric standard deviation) in the children was 1.53 (1.57) μg/dL. The highest, median, and lowest blood lead levels were 4.89, 1.58, and 0.11 μg/dL, respectively. In our study, 25.6% (*n* = 400) of the children had blood lead levels of >2.0 μg/dL.

**Fig. 1:**
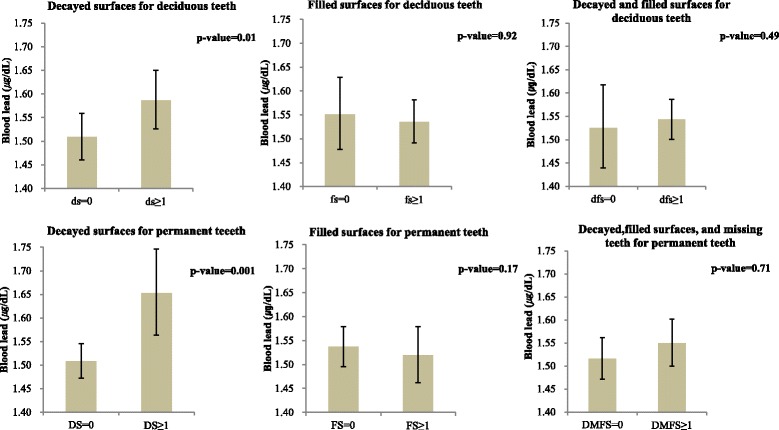
Blood lead concentration according to dental caries status. Blood lead concentrations were estimated with the least-square mean by using log-transformed and exponential function, adjusted for gender, age, educational level of mother, and urinary cotinine

The least square mean blood lead level adjusted for potential confounding factors was significantly higher in the children with than in those without decayed surfaces in both deciduous and permanent teeth: 1.59 μg/dL (95% CI: 1.53–1.65 μg/dL) versus 1.51 μg/dL (1.46–1.56 μg/dL) for deciduous teeth (*p* = 0.01); and 1.65 μg/dL (1.56–1.75 μg/dL) versus 1.51 μg/dL (1.47–1.55 μg/dL) for permanent teeth (*p* = 0.001). However, the blood lead levels were not different between the children with filled surfaces, missing teeth, or both and those with neither filled surfaces nor missing teeth in both deciduous and permanent teeth (Fig. 1).

The prevalence ratio and 95% CI of the risk of having dfs increased with increasing blood lead concentrations in a dose-dependent manner (1.14, 1.02–1.27). However, permanent teeth were negatively associated with higher blood lead levels (0.83, 0.69-0.99; Table 2).

**Table 2:** Association between blood lead concentration and dental caries in deciduous and permanent teeth

		Crude model		Adjusted model^a^		
For Blood lead level (ug/dL)	N(≥1)	Prevalence Ratio	95% CI	Prevalence Ratio	95% CI	Model fit (*p*-value)^*^
Deciduous teeth						
ds	520	1.15	0.96–1.36	1.16	0.91–1.49	22.86 (0.0018)
fs	903	1.08	0.98–1.20	1.11	0.98–1.25	25.70 (0.0006)
dfs	1,014	1.09	1.00–1.19	1.14	1.02–1.27	35.09 (<.0001)
Permanent teeth						
DS	255	0.77	0.62–0.97	0.69	0.45–1.07	9.87 (0.1960)
FS	523	0.89	0.81–0.99	0.87	0.73–1.04	16.25 (0.0229)
DMFS	698	0.86	0.78–0.95	0.83	0.69–0.99	14.56 (0.0421)

Prevalence ratio and 95% Confidence Intervals (CI) estimated using zero inflated negative binomial (ZINB) model

*ds* decayed surfaces for deciduous tooth, *fs* filled surfaces because of caries for deciduous tooth, *dfs* the sum of decayed and filled surfaces for deciduous tooth. *DS* decayed surfaces for permanent tooth, *FS* filled surfaces because of caries for permanent tooth, *DMFS* the sum of decayed, filled surfaces and missing because of caries for permanent tooth

^*^Chi-square difference values for likelihood ratio test

^a^ZINB model adjusted for gender, age (categorical), household income (categorical), and urinary cotinine level (categorical)

## Discussion

The results of this study show a significant increased risk of dental caries with increasing blood lead level among children with blood lead levels of <5 μg/dL, particularly for deciduous tooth surfaces, which exhibited a linear relationship.

The mean blood lead concentration in our study subjects (GM, 1.53 μg/dL; maximum, 4.89 μg/dL) was lower than those in previous reports of a significant association between blood lead and dental caries. The mean blood lead levels in these reports were between 2.1 (0.10 μmol/L) and 2.8 μg/dL (0.14 μmol/L) [18], specifically 10.7 μg/dL [19], 2.3 μg/dL [20], 7.2 μg/dL [21], and 4.6 μg/dL [22]. All these blood lead levels were higher than those in the present study (<5 μg/dL), which was recommended recently as the reference value by the US CDC to protect children [23].

The significant relationship in deciduous teeth is consistent with all of the above-mentioned epidemiological studies [19–22], except one study that reported a significant association in permanent teeth [18]. One possible explanation for the difference is that the subjects in the previous study (2–17 years old) [18] were older than those in other studies, including the present study, in which sufficient time for development of caries (DMFS) in permanent teeth was allowed. A recent study using the ZINB model also showed a significant association between blood lead and dfs, which is consistent with our findings [27].

We also found significantly higher blood lead levels in the children with decayed tooth surfaces but found no significant differences between the children with and those without filled or both decayed and filled surfaces in both deciduous and permanent teeth. The decayed surfaces reflect currently extant and not-yet-treated caries, while filled surfaces are developed and treated caries, and reflect the availability of oral health-care services. Therefore, decayed surfaces might be a better indicator of current lead exposure related dental caries.

For permanent dental caries, DMFS showed a significant negative association with blood lead level. Unlike deciduous teeth, which are temporary for a certain period and soon replaced by permanent teeth, permanent teeth might have attracted more attention from parents and received more care services. Therefore, the higher prevalence of DMFS in permanent tooth than caries in deciduous tooth is more influenced by other factors such as social and health-care conditions rather than causal risk factors such as lead exposure. In this case, the reverse causality between blood lead level and permanent dental caries might be a more plausible explanation considering the cross-sectional design of the present study. As lead level has been reported to be higher in those with lower socioeconomic position (SEP) in this population [29], children with higher SEP, well-treated caries, and more DMFS showed lower blood lead level.

Although the adjusted mean blood lead level was significantly higher in the children with than in those without dental caries both in deciduous and permanent teeth, the significant increase in the risk of having dental caries was found only in deciduous teeth in the present study. The difference might be due in part to the different statistical models for estimating adjusted mean and risk of having caries. However, an important thing might be the susceptibility to lead toxicity in caries development because younger children have deciduous teeth rather than permanent teeth.

A previous study [22] reported a sex-related difference in the association between blood lead and dental caries; that is, a relationship existed only in males. We thus reanalyzed the data with regard to sex. However, we could not reproduce the finding of the previous study and found no significant difference between the sexes (prevalence ratio [95% CI] for dfs: 1.11 [1.00–1.23] in males and 1.07 [0.94–1.22] in females, p value for multiplicative interaction for sex = 0.60).

Lead is initially distributed to soft tissues such as the kidney and liver, and then redistributed to bone and hair [10]. Lead is absorbed into the teeth and delays the mineralization of enamel [19]. In blood, lead has a half-life of up to 30 days and is excreted into the urine; it can be stored in bone for >20 years [30]. Persistent release of lead from bone into the blood can delay tooth calcification, in which the mineralization process continues even after oral tooth eruption. Dental caries can be prevented by regular brushing and water fluoridation. However, lead may bind to fluoride ions in saliva and plaque, and thereby reduce the ability of fluoride to remineralize enamel after an acid challenge [31].

This study has several limitations. First, lead exposure at the time of enamel formation is the mechanism most relevant to a causal lead-caries association [18]. This is a cross-sectional study, so we could not provide evidence that the teeth were influenced by blood lead level during the period of ontogeny or after mineralization. To identify a causal relationship between blood lead level and dental caries, a cohort study that measures lead exposure in pregnant women and then observes their infants is warranted. Next, we did not include calcium and iron intakes in our multivariate models as covariates that influence bone and teeth growth. However, the present results may not be confounded by these factors because blood lead levels were not correlated with calcium or iron concentrations in the present study.

In conclusion, we found a linear dose–response association between low blood lead levels (<5 μg/dL) and the development of caries in deciduous teeth.

## Conclusions

This study showed an association between low blood lead levels (<5 μg/dL) and development of dental caries in deciduous teeth but not in permanent teeth. These results suggest that blood lead level is a risk factor for the development of dental caries in deciduous dentition.

## Abbreviations

CDC: Centers for disease control and prevention
CHEER: Children’s Health and Environment Research
CI: Confidence interval
dfs: The sum of decayed and filled surfaces for deciduous teeth
DMFS: The sum of decayed and filled surfaces and missing teeth due to dental caries for permanent teeth
ds: The number of decayed surfaces for deciduous teeth
DS: The number of decayed surfaces for permanent teeth
FS: The number of filled surfaces for permanent teeth
fs: The number of filled surfaces for deciduous teeth
IQ: Intelligence quotient
LOD: Limit of detection
LOQ: Limit of quantification
SEP: Socioeconomic position
ZINB: Zero-inflated negative binomial model
